# Retraction Note: Exposure to pesticide components causes recurrent pregnancy loss by increasing placental oxidative stress and apoptosis: a case–control study

**DOI:** 10.1038/s41598-023-46570-6

**Published:** 2023-11-07

**Authors:** Mona A. H. El-Baz, Ahmed F. Amin, Khalid M. Mohany

**Affiliations:** 1 Medical Biochemistry and Molecular Biology Department, Faculty of Medicine, Assiut University, EL Gammaa Street, Assiut City, 71515 Egypt; 2 Department of Obstetrics and Gynecology, Faculty of Medicine, Women Health Hospital, Assiut University, Assiut City, 71515 Egypt

Retraction of: *Scientific Reports* https://doi.org/10.1038/s41598-023-36363-2, published online 05 June 2023

The Editors have retracted this Article.

After publication, it was brought to the Editors’ attention that the methods used to determine the sensitivity, specificity, and cut-off values presented in Table 5 are not described in sufficient detail to allow reproduction of the analyses. The Editors contacted the Authors to request clarifications and the raw data underlying the study. The Authors were not able to provide a clear and valid description of the methodology. They did provide some of the data; however, these were not the original raw data and the Editors were not able to confirm their veracity.

The Editors therefore no longer have confidence in the results and conclusions presented in this Article.

Khalid M. Mohany disagrees with the retraction. Mona A. H. El-Baz and Ahmed F. Amin did not respond to the correspondence about this retraction.

